# The PJI-TNM Classification as Predictor for Revision-Free Implant Survival Rates in Patients with Periprosthetic Joint Infection of the Hip or Knee Joint

**DOI:** 10.3390/idr17030054

**Published:** 2025-05-15

**Authors:** Frank Sebastian Fröschen, Lisa Greber, Ernst Molitor, Gunnar Thorben Rembert Hischebeth, Alexander Franz, Thomas Martin Randau

**Affiliations:** 1 Department of Orthopaedics and Trauma Surgery, University Hospital Bonn, 53127 Bonn, Germany; s4lagreb@uni-bonn.de (L.G.); alexander.franz@ukbonn.de (A.F.); trandau@severinskloesterchen.de (T.M.R.); 2 Institute of Medical Microbiology, Immunology and Parasitology, University Hospital Bonn, 53127 Bonn, Germany; molitor@uni-bonn.de (E.M.); hischebeth@microbiology-bonn.de (G.T.R.H.); 3 Department of Trauma and Orthopedic Surgery, BG Klinik Ludwigshafen, 67071 Ludwigshafen, Germany; 4 Clinic for Orthopedics, Special Orthopedic Surgery and Sports Medicine, Augustinian Sisters Hospital, 51109 Cologne, Germany

**Keywords:** arthroplasty, periprosthetic joint infection, PJI-TNM classification, orthopaedic infections

## Abstract

Background: Periprosthetic joint infections (PJIs) remain a major challenge in arthroplasty. This study tries to evaluate the PJI-TNM classification as predictor for the revision-free implant survival in patients with PJI of the hip or knee joint. Methods: To this end, we perform a retrospective study of all consecutive patients with PJI of an inlying hip or knee arthroplasty between January 2015 and December 2019. Results: A total of 443 cases (hip: *n* = 247; knee *n* = 196) were identified. In total, 439 patients underwent surgery (DAIR: *n* = 138 cases (31%), explantation: *n* = 272 (61%), irrigation with debridement without exchange of implant components: *n* = 29 (6.5%)). Four patients refused surgical treatment and 39.5% were lost to follow-up. In total, 78 patients died during follow-up and 27 deaths were directly related to PJI/complications during treatment. Patients with inlying “standard”-implants (*p* < 0.001) and without previous history of PJI (*p* = 0.002) displayed a significantly higher postoperative revision-free implant survival. In terms of the PJI-TNM subclassification, patients with loosened implants but without soft-tissue defects (T1) displayed the highest revision-free implant survival. In contrast, patients classified as M3 (no surgical treatment possible) displayed an inferior outcome compared to M0, M1, or M2. Patients with different N-subclassifications (“non-human cells”/causative pathogen) did not display differences in revision-free implant survival. Conclusions: The PJI-TNM classification is well suited to classify PJIs. Its complexity allows for more than 500 different combinations of classifications. Further validation data are needed, but to us, the PJI-TNM classification seems to offer the possibility of comparing patients with PJIs. It may, therefore, be a very valuable tool in order to compare cohorts with PJIs and provide individual data for patient specific outcomes.

## 1. Introduction

Periprosthetic joint infections (PJIs) after primary or revision arthroplasty remain the most feared and relevant complication in orthopaedic surgery [1]. High complications and reinfection rates remain a challenge, despite ongoing advances in the field of preoperative antibiotic prophylaxis, less invasive surgical techniques, coated implants, and postoperative antibiotic therapy [2,3]. From a surgical point of view, curative treatment options include a DAIR-procedure (Debridement, Antibiotics, Irrigation, Retention of implants) in patients with well-fixed implants and a short duration of symptoms, or, in patients with a chronic infection with or without a loosened implant, an explantation of the inlying implant with a subsequent one-stage or two-stage procedure [4,5]. The DAIR procedure is characterised by surgical debridement of the surrounding infected tissue and an exchange of the mobile parts of the inlying implants followed by irrigation and postoperative antibiotic therapy. The well-fixed prosthetic components may be retained, but DAIR should not be performed in chronic PJIs [6]. It is recommended to perform DAIR procedures only in patients with acute PJIs, where biofilm formation is thought to be in its initial stages and, therefore, more susceptible to eradication [7,8]. Otherwise a two-stage exchange remains the gold-standard for treatment of chronic PJI, although one-stage exchanges have shown promising results [9].

Deciding the right treatment option is a complex process and must take into account several individual factors, including inlying implant or morbidity of the patient. The overall treatment failure rates for reinfection, according to the recent annual reports of arthroplasty registers, are often presented to the patient in the process of shared-decision making [10]. These failure rates often do not consider the combination of an inlying implant, host-related factors such as comorbidities, and pathogen-related aspects. In short, they do not consider individual aspects at the time of diagnosing a PJI. Therefore, they lack the only relevant information patients want to know from their responsible orthopaedic surgeon: the outcome for a patient “like me”. Furthermore, a classification should always provide new insides for healthcare specialists (e.g., risk of revision surgery).

To optimise these individual aspects, Alt. et al. suggested a new classification system, the PJI-TNM classification, which tries to consider implant-, host-, and pathogen-related aspects of patients with PJI [11].

The analogy to the well-known TNM classification is, in this context, fully intentional [11]. Whereas the oncological classification is based on the tumour characteristics/expansion, infiltration of lymphatic nodes, and remote metastasis (TNM), in PJI, the letters T, N, and M consider the condition and type of the implant (“Tissue and implant condition”: T), the causative pathogen (“Non-human cells”: N), and host-related variables (“Morbidity”: M) with the additional option to classify patients who had a reinfection (“r”) [12].

While it seems confusing at first to align the classification of PJI with that of malignant diseases, this analogy is fully intentional. Recent studies report 5-year mortality rates for PJI of up to 21%, which is comparable to malignant diseases [13,14]. The PJI classification was created as a reminder for practitioners of the often chronic and highly “malignant” nature of PJI.

To date, the PJI-TNM classification has been in use for the classification of PJIs in small cohorts [12,15]. Nevertheless, data evaluating the revision-free implant survival for each PJI-TNM stage are still missing. The aim of this study was to evaluate the usefulness of the PJI-TNM classification in predicting patient-specific outcomes for PJI. To this end, we retrospectively analysed data from a large cohort under the end-point of revision-free survival. Additionally, we evaluated whether implant survival rates differed among different subclassifications, with the aim of helping orthopaedic surgeons better predict the likely outcomes for patients throughout all constellations of the classification system.

## 2. Materials and Methods

In this retrospective study, we included all consecutive cases with PJI of the hip or knee joint at a tertiary endoprosthetic referral centre in Germany between January 2015 and December 2019. The study was approved by our local institutional ethic board (University of Bonn Ethics Committee, No. 199/22). Due to the study’s retrospective nature, informed consent was not required.

A PJI was defined according to Parvizi et al., with patients fulfilling one of the following criteria: (1) presence of a sinus tract communicating with the prosthesis, (2) isolation of the same microorganism from two or more cultures/tissue biopsies obtained from the infected joint, or (3) isolation of one microorganism in the intraoperative cultures with additional evidence of an infection of the inlying implant (positive histology, presence of purulence, elevated serum erythrocyte sedimentation rate, elevated C-reactive protein, and elevated synovial white blood cell count) [16].

In order to better characterise our patient population, we recorded patient demographics, weight, site, and type of arthroplasty, previous history, comorbidities, performed procedures (debridement, antibiotics, irrigation, and implant retention [DAIR]; one-stage or two-stage exchange; debridement without changing parts of the inlying implants), surgery time (incision to suture time), and laboratory parameters. Debridement without an exchange of any parts of the inlying implants was only performed if the required implants were not available or if the patient refused to have implant components exchanged. In patients undergoing DAIR, an exchange of all mobile parts was routinely performed. This includes exchanging, e.g., bearings and modular parts of revision implants not connected to the bone (e.g., proximal or distal femoral replacement).

We differentiated between acute (symptom duration < 3 weeks) and chronic infections (symptom duration > 3 weeks) following an adapted version of the Tsukayama and Izakovicova classification [8,17]. Therefore, DAIR was not performed in patients with chronic PJIs, patients with a sinus tract communicating with the prosthesis, or patients with a loosening of the infected inlying implant. All cases were classified according to the PJI-TNM classification system (Table 1) [11,12,15].

In addition, postoperative follow-up after last surgery was assessed in order to evaluate implant survival. In detail, clinical and radiographic follow-up assessments (X-ray-imaging in 2 planes) are normally performed postoperatively at 6 weeks, 6 months, and 1 year, and, thereafter, in 1 year intervals. Postoperative radiological images were evaluated for new osteolysis, significant or progressive radiolucent lines as compared to previous images, and migration of the acetabular, femoral, or tibial prosthesis component.

Implant survival was defined as a lack of clinical signs of a renewed infection and the absence of any reason for operative revision at last follow-up. In patients in need of a two-stage exchange, follow-up started with the implantation of the “new” implant and, therefore, when the infection was assumed to have been eradicated. Antibiotic therapy was performed for 6 weeks after DAIR and up to 6 weeks after a two stage exchange according to the results of the antibiotic susceptibility testing.

Furthermore, an evaluation of all microbiologically detected causative pathogens in specimens (tissue biopsies, synovial fluid, and sonication fluids of explanted prostheses) obtained intraoperatively was performed.

**Figure 1:**
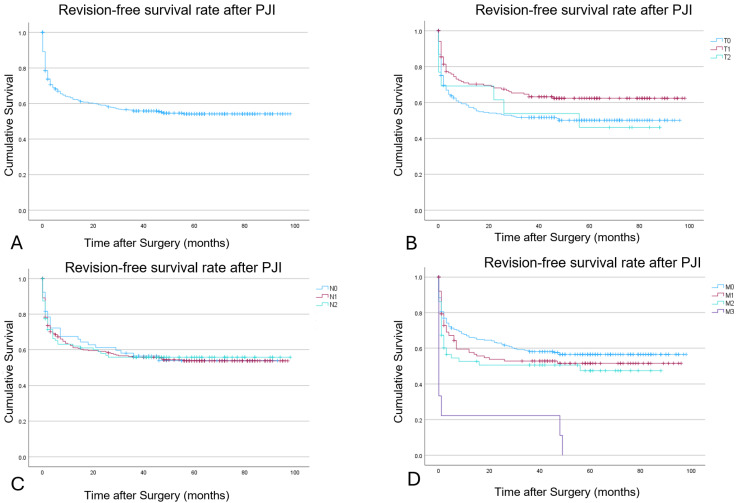
Kaplan–Meier survivorship with the endpoint need for revision surgery ((**A**) overall; (**B**) for T subtypes; (**C**) for N subtypes; (**D**) for M subtypes).

**Figure 2:**
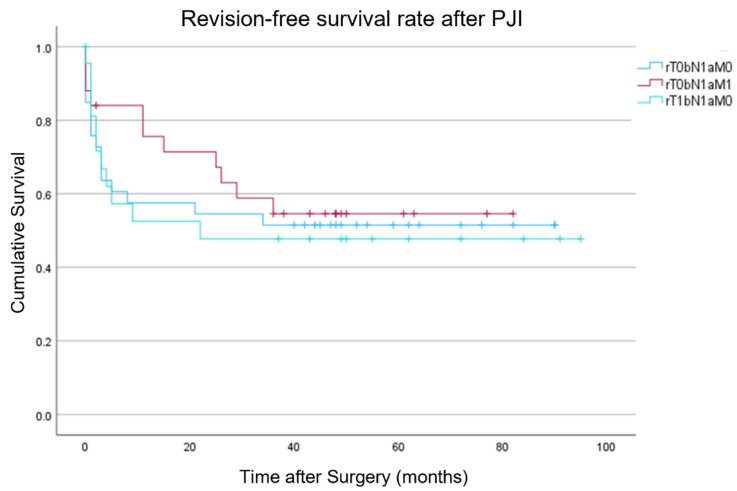
Kaplan–Meier survivorship with the endpoint need for revision surgery the three most frequent PJI-TNM subtypes (rT0bN1aM0, rT0bN1aM1, and rT1bN1aM0).

**Figure 3:**
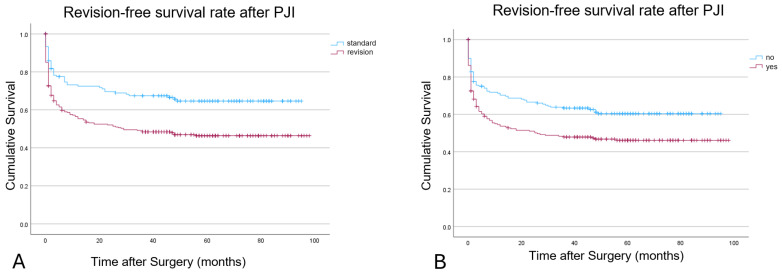
Kaplan–Meier survivorship with the endpoint need for revision surgery for an inlying implant (**A**) and previous history of PJI (**B**).

Statistical analysis: Data were collected in Microsoft Excel 2022 (Microsoft Corporation, Redmond, WA, USA). Statistical analysis was carried out using SPSS statistics 29 for Windows (SPSS, Inc., an IBM company, Chicago, IL, USA). Descriptive statistics, including arithmetic mean values and standard deviations, were calculated. Data are given as means ± standard deviation (SD). To analyse categorial data, Pearson’s chi-squared test was used to test for an association. The Shapiro–Wilk test was used to verify the normal distribution of continuous variables. For continuous variables, a two-tailored *t*-test or the Mann–Whitney U test was performed. A *p*-value less than 0.05 was considered statistically significant. A Kaplan–Meier survival analysis with the endpoint need for revision surgery (Figures 1–3) was calculated. To access differences between subgroups in the survival analysis, the log-rank test was used (*p* < 0.05).

## 3. Results

### 3.1. Patient Demographics and Infection Characteristics

A total of 443 consecutive cases with acute or chronic PJI of the hip or knee joint were identified and evaluated. No case had to be exclude, because of missing/incomplete data needed for classification according to PJI-TNM. For demographic data, see Table 2; for implant-related data, see Table 3; and for comorbidities, see Table 4. In 200 (74%) of those 272 patients in need of an explantation of the inlying implant, reimplantation was performed.

### 3.2. Classification According to the PJI-TNM Classification

All 443 patients could be classified according to the PJI-TNM classification. For Details, see Table 5. In total, 240 patients (54.2%) had a previous history of PJI and were therefore classified as “r”. The most frequent PJI-TNM classifications in our cohort were rT0bN1aM0 (*n* = 33; 7%), rT0bN1aM1 (*n* = 25; 6%), and rT1bN1aM0 (*n* = 22; 5%). The most frequent category for T, N, and M were T0b (41.3% of all T subtypes), N1a (44% of all N subtypes), and M0 (59.1% of all M subtypes).

### 3.3. Outcome Evaluation

The mean follow-up after last revision-surgery of all patients was 30 ± 26 months. Only 60.5% (*n* = 268) presented for postoperative follow-up assessment at 6 months or later, despite our best efforts to contact them. Therefore, 39.5% were lost to follow-up. Out of these 268 patients, which presented to postoperative follow-ups, 29.2% (*n* = 82) were considered clinically successful with no sign of a renewed infection or need for revision surgery at last follow-up. In contrast, 70.8% (*n* = 190) needed revision surgery. Revision-free follow-up of patients, which used our follow-up assessments, was 35 ± 31 months. In total, 78 patients died during follow-up, and only 27 of these 78 cases (*n* = 34.6%) were directly related to the PJI/complications during treatment.

A Kaplan–Meier survival analysis was performed to access the overall revision-free survival rate, as well as the survival rate for the different subtypes for T, N, and M (Figure 1). In detail, the overall revision-free survival rate was 62%, 58%, and 55% at 12, 24, and 48 months. The Survival rates for the T, N, and M subtypes at 12, 24, and 48 months are displayed in Table 6.

The log-rank test did show a statistically significant higher survival rate for patients with T1 in comparison with T0 or T2 (*p*: 0.032). For N-subtypes, there was no statistically significant difference in revision-free implant survival for the different subtypes in comparison to each other (*p*: >0.05). For the M-subtypes, patients with M3 had a significantly worse survival rate (*p*: <0.001). The difference between M0, M1, and M2 was not statistically significant.

Figure 2 displays Kaplan–Meier survivorship with the endpoint need for revision surgery for the three most frequent PJI-TNM subtypes (rT0bN1aM0, rT0bN1aM1, rT1bN1aM0) in our cohort. There was no statistically significant difference in revision-free implant survival at 48 months (*p*: >0.05).

For the “r” subtype, we could detect a significantly (*p*: 0.002) worse revision-free implant survival in patients with a previous history of PJI (Table 6; Figure 3). Patients with inlying “standard” implants displayed a significantly (*p*: <0.001) higher revision-free implant survival (12 months: 72% vs. 55%; 24 months: 69% vs. 52%; 48 months: 65% vs. 46%).

### 3.4. Microbiological and Histopathological Evaluation

Detection of a causative pathogen from an intraoperative specimen or preoperatively acquired joint fluid was possible in 320 cases (72%). In 80 patients (18%), two or more causative pathogens could be detected. In summary, 331 different pathogens could be detected. The most frequently detected pathogens were coagulase-negative staphylococci (*n* = 121 (36.55%)), *S. aureus* (*n* = 66 (19.93%)), Streptococci spp. (*n* = 47 (14.19%)), and *E. coli* (*n* = 25 (7.55%). In 11% (*n* = 50) of all cases, a pathogen could be classified as “difficult-to-treat”. A list of all detected pathogens is provided in Appendix A (Table A1).

## 4. Discussion

The most serious complication after primary or revision arthroplasty remains a PJI. In order to choose the best clinical treatment pathway, a practical and reliable classification is needed.

However, most clinicians still use well known “simple” classifications and distinguish between early and late PJIs in combination with the onset (acute vs. chronic) of symptoms [7]. Commonly, a short duration of symptoms is associated with a better outcome [20]. Here, the cut-off value of an acute PJI in weeks ranges between 0 and 12 weeks for symptom duration [7,17]. For treatment, this differentiation remains the most relevant. Patients with an acute PJI and without loosening of the inlying components undergo surgical debridement with an exchange of the “mobile parts”, but without removing the well-fixed inlying implant (DAIR-procedure (Debridement, Antibiotics, Irrigation, and implant Retention) [21]. In contrast, patients with chronic PJIs undergo revision surgery with an exchange of the inlying implant. To date, the two-stage exchange remains the gold standard, although one-stage procedures show promising results [22].

As PJIs are heterogeneous and complex, these “simple” classifications might be too superficial and do not provide any detailed predictive possibilities. Therefore, McPherson et al. were the first to propose a more advanced classification incorporating three components: infection type, systemic host grade, and local extremity grade [23,24].

The classification was based on the authors’ experience and local criteria without considering the inlying implant or the causative pathogen. To improve treatment, more comprehensive, memorable, structured, and established classifications might be useful. The PJI-TNM classification published by Alt et al. tries to address this point to improve treatment; however, it was initially evaluated in 20 patients only [11,12]. Although intra- and interobserver variability have already been examined with very good results where intraoperative findings are concerned [15], larger studies evaluating the PJI-TNM classification do not exist. Our most important finding is that we were retrospectively able to classify all patients in need of treatment for PJI at a tertiary endoprosthetic referral centre. Therefore, this complex classification system is well suited for daily routine. The previously observed difficulty in using the PJI-TNM classification for differentiating between a stable and loose implant could be avoided in our cohort, as the intraoperative findings in combination with the radiological findings were used for classification [15].

Another topic of ongoing debate might be the definition of treatment failure after PJI in (revision) arthroplasty. We decided to define treatment failure as the necessity for revision surgery because of a reinfection or persisting infection. Here, the risk for reinfection and the impact on patients’ mental health due to immobility during a two stage exchange are well known [25]. Therefore, we are in dire need of the best possible preoperative “preparation” for surgeon and patient, which may be achieved by a better understanding of the specific revision-free survival rates for individual cases. For overall comparison: our overall revision-free implant survival rate of 62%, 58%, and 55% at 12, 24, and 48 months, respectively, is lower than the 80% at 48 months postoperative reported for revision hip arthroplasties in previous studies, e.g., by Vasarhelyi et al. [26]. Nevertheless, it remains comparable to a report of revision-free implant survival of 55% by Lim et al. at 3 years, or the 57% reported by Chalmers et al. at 5 years for knee arthroplasty [27,28]. Here, the PJI-TNM classification offers a unique advantage, as it is the only classification system that allows for a comparison of revision-free implant survival under the aspects of pathogen, host status, and inlying implant. In this context, numerous previous studies tried to define host-related factors for PJI [29]. Coagulopathy, preoperative anaemia, congestive heart failure, chronic pulmonary disease, depression, renal disease, pulmonary circulation disorders, psychoses, metastatic tumour, peripheral vascular disease, and valvular disease all were evaluated as risk factors for PJI [30]. Nevertheless, the knowledge of risk factors in general might not be sufficient when it comes to providing the best possible care for an individual patient, or in order to educate a patient on their expected implant survival. Therefore, a unique advantage of the PJI-TNM is its complexity, as this complexity allows for the classification of patients in detail while providing a detailed revision-free implant survival (rate) on an individual basis. With more than 500 different possible PJI-TNM classification options, we were not able to provide large cohorts for all combinations. According to our data, loosened implants were associated with a higher implant survival rate compared to stable implants with or without soft tissue defects. A possible explanation might be that loose implant allow for an easier explantation.

In contrast, neither the causative pathogen nor the morbidity of the patients taken on their own had a significant effect on the revision-free implant survival rate. Here, we would like to highlight that our pathogen distribution (N-classification) did not differ from those reported in the literature for PJIs [1,31,32,33,34]. In this context, we would like to point out that the graph for M0 shows a higher survival rate than M1 or M2; however, this was not statistically significant. These two observations might be interesting, as PJIs with “difficult-to-treat pathogens” and PIJs in multimorbid patients are, in general, associated with a worse outcome [35,36,37]. Although the PJI-TNM classification considers several factors, it does not consider the immune status of the patients. Piuzzi et al. outlined the role of the immune system in this context. They suggested that a preoperative immune system assessment might help to optimise treatment in patients with PJI [38].

A previous history of PJI has been reported as a relevant risk factor for renewed PJI and low revision-free implant survival in both studies and recent annual reports of arthroplasty registers [39]. Our data support this. There is a significant difference in patients with and without a previous history of PJI. Patients with “first-time” PJI displayed an acceptable revision-free implant survival at 4 years of 61%.

The present study has several potential limitations. Our data were collected at a tertiary endoprosthetic referral centre, where patients are often transferred to in case of complications (e.g., persisting or recurrent PJI, or patient-related factors such as multimorbidity), which manifests in the high number of patients classified as “r”. Here, additional studies involving several endoprosthetic referral centres might be helpful. The high percentage of patients lost to follow-up and death might have further influenced our results. In addition, we were not able to provide sufficiently large subgroups of patients for all possible combinations of the PJI-TNM classification system.

## 5. Conclusions

The complexity of the PJI-TNM classification corresponds very well with the heterogeneous characteristics of patients with PJI. All facets of a PJI, such as inlying implants, causative pathogens, or host-related factors (morbidity), can be classified with the help of the PJI-TNM classification. Although not all subclassifications showed significant differences in the revision-free implant survival rate, the PJI-TNM classification might be a valuable tool for providing orthopaedic surgeons with patient specific outcomes. Further studies are needed for an in-deep evaluation.

## Figures and Tables

**Table 1:** The PJI-TNM classification by Alt et al. [11,18].

T-tissue and implant conditions			
	T0	a	Stable standard implant without important soft tissue defect
		b	Stable revision implant without important soft tissue defect
	T1	a	Loosened standard implant without important soft tissue defect
		b	Loosened revision implant without important soft tissue defect
	T2	a	Severe soft tissue defect with standard implant
		b	Severe soft tissue defect with revision implant
N-non-human cells			
	N0	a	No mature biofilm formation (former: acute), directly postoperatively
		b	No mature biofilm formation (former: acute), late hematogenous
	N1	a	Mature biofilm formation (former: chronic) without “difficult-to-treat bacteria”
		b	Mature biofilm formation (former: chronic) with culture-negative infection
	N2	a	Mature biofilm formation (former: chronic) with “difficult-to-treat bacteria”
		b	Mature biofilm formation (former: chronic) with polymicrobial infection
		c	Mature biofilm formation (former: chronic) with fungi
M-morbidity of the patient			
	M0		Not or only mildly compromised (Charlson comorbidity index: 0–1)
	M1		Moderately compromised patient (Charlson comorbidity index: 2–3)
	M2		Severely compromised patient (Charlson comorbidity index 4–5)
	M3	a	Patient refuses surgical treatment
		b	Patient does not benefit from surgical treatment
		c	Patient does not survive surgical treatment
r—reinfection			If the infection involves a previously infected implant, the situation is considered to be “reinfection” and an “r” is placed in front of the classification

**Table 2:** Descriptive summary of the clinical and laboratory data at admission. BMI: body-mass-index; WBC: white blood cell count; PMN: polymorphonuclear cell (neutrophil); M: male; F: female.

Clinical Features	2015–2019
Number of patients	443 (222 M; 221 F)
Age (range) [years]	70.5 ± 11.9 (21–98)
BMI (range) [kg/m^2^]	29.5 ± 6.9 (14.2–60.6)
Hospital stay (range) [days]	26.1 ± 25.5 (2–354)
Infected joint: hip/knee	247 (55.8%)/196 (44.2%)
Preoperative C-reactive protein [mg/L]	71.3 ± 87.1 (0.5–557.6)
Preoperative leukocyte count [G/L]	9.4 ± 5.0 (2.7–56.2)
Preoperative haemoglobin [g/dL]	11.5 ± 2.2 (6.7–18.7)
Preoperative Creatinine [mg/dL]	1.0 ± 0.5 (0.2–5.3)
Preoperative synovial cell count [cells/µL] (*n* = 180) Total cell count/µL WBC count/µL PMN count/µL	67,942.2 ± 122,785.9 66,216.3 ± 119,306.6 48,439.4 ± 79,964.9

**Table 3:** Implant-related data and performed procedure. DAIR: Debridement, Antibiotics, Irrigation, and Implant retention.

PJI-TNM Classification	2015–2019
Inlying implant ad first admission: standard implant/revision implant	150 (33.9%)/293 (66.1%)
Standing time implant at PJI [months]	23.7 ± 33.1 (0–155)
Performed operative procedure: DAIR Debridement without implant revision Explantation Refusal of needed operative treatment	138 (31%) 29 (7%) 272 (61%) 4 (1%)
Incision to suture time [min]	149.9 ± 63.5 (26–348)

**Table 4:** Comorbidities classified according to Charlson et al. [19]. AIDS: acquired immune deficiency syndrome.

Comorbidities	Hip (*n*/%)	Knee (*n*/%)	Total (*n*)
Myocardial infarction	29 (11.7%)	24 (12.2%)	53 (23.9%)
Congestive heart failure	39 (15.8%)	42 (21.4%)	81 (37.2%)
Peripheral vascular	9 (3.6%)	9 (4.6%)	18 (8.2%)
Deep vein thrombosis/pulmonary embolism	21 (8.5%)	20 (10.2%)	41 (18.7%)
Cerebrovascular	15 (6.1%)	16 (8.2%)	31 (14.3%)
Hemiplegia	2 (0.8%)	0 (0.0%)	2 (0.8%)
Dementia	5 (2.0%)	5 (2.6%)	10 (4.6%)
Pulmonary	40 (16.2%)	43 (21.9%)	83 (38.1%)
Collagenosis	28 (11.3%)	19 (9.7%)	47 (21%)
Peptic ulcer	22 (8.9%)	17 (8.7%)	39 (17.6%)
Diabetes with/without end organ damage	47 (19.0%)	48 (24.5%)	95 (43.5%)
Liver damage	14 (5.6%)	8 (4.0%)	22 (9.6%)
Cancer	20 (8.1%)	13 (6.6%)	33 (14.7%)
AIDS	1 (0.4%)	1 (0.5%)	2 (0.9%)

**Table 5:** Subclassification of all included patients according to PJI-TNM (ntotal = 443).

PJI-TNM Classification	2015–2019
Tissue and implant conditions	
T0a/T0b	87 (19.6%)/183 (41.3%)
T1a/T1b	60 (13.5%)/99 (22.3%)
T2a/T2b	5 (1.1%)/9 (2.0%)
Non-human cells	
N0a/N0b	29 (6.5%)/38 (8.6%)
N1a/N1b	195 (44.0%)/76 (17.2%)
N2a/N2b/N2c	33 (7.4%)/65 (14.7%)/7 (1.6%)
Morbidity	
M0	262 (59.1%)
M1	114 (25.7%)
M2	58 (13.1%)
M3a/M3b/M3c	4 (0.9%)/0 (0%)/5 (1.1%)

**Table 6:** Kaplan–Meier survivorship with the endpoint need for revision surgery at 12, 24, and 48 months for each subtype, “r”, and the three most common classifications.

Tissue and Implant Conditions	12 Months	24 Months	48 Months
T0	56%	53%	41%
T1	70%	67%	55%
T2	69%	54%	46%
Non-human cells			
N0	66%	61%	40%
N1	60%	58%	50%
N2	61%	55%	45%
Morbidity			
M0	66.1%	62.0%	56.5%
M1	57.5%	52.7%	51.4%
M2	52.5%	50.5%	50%
M3	22.2%	22.2%	11.1%
Overall			
rT0bN1aM0	58%	52%	41%
rT0bN1aM1	75%	67%	42%
rT1bN1aM0	52%	48%	39%
Reinfection			
“r”	53%	50%	46%
Non-“r”	70%	66%	61%

## Data Availability

The original contributions presented in this study are included in the article; further inquiries can be directed to the corresponding author.

## Appendix A

**Table A1:** Culture data from preoperative joint aspirate (synovial fluid), sonication fluid, and intraoperative specimen.

Culture Data	Total by Species	
*Staphylococcus aureus*	66	19.94%
*Staphylococcus epidermidis*	86	25.98%
*Staphylococcus haemolyticus*	6	1.81%
*Staphylococcus hominis*	8	2.42%
*Staphylococcus lugdunensis*	14	4.23%
*Staphylococcus mitis*	1	0.30%
*Staphylococcus pasteuri*	1	0.30%
*Staphylococcus saprophyticus*	1	0.30%
*Staphylococcus warnerii*	4	1.21%
*Streptococcus agalactiae*	12	3.63%
*Streptococcus anginosus*	4	1.21%
*Streptococcus dysgalactiae*	8	2.42%
*Streptococcus gordonii*	1	0.30%
*Streptococcus intermedius*	1	0.30%
*Streptococcus mitis*	8	2.42%
*Streptococcus oralis*	1	0.30%
*Streptococcus parasanguinis*	2	0.60%
*Streptococcus pneumoniae*	1	0.30%
*Streptococcus pyogenes*	3	0.91%
*Streptococcus salivarius*	2	0.60%
*Streptococcus Salivarius*	4	1.21%
*Bacillus licheniformis*	1	0.30%
*Bacillus simplex*	1	0.30%
*Brevibacterium luteolum*	1	0.30%
*Corynebacterium aurimucosum*	1	0.30%
*Corynebacterium striatum*	1	0.30%
*Corynebacterium urealyticum*	1	0.30%
*Dermabacter hominis*	1	0.30%
*Erysipelothrix rhusiopathiae*	1	0.30%
*Kocuria kristinae*	1	0.30%
*Micrococcus luteus*	4	1.21%
*Propionibacterium acnes*	14	4.23%
*Acitenobacter baumanii*	1	0.30%
*Bacteroides fragilis*	2	0.60%
*Burkholderia species*	1	0.30%
*Enterobacter cloacae*	5	1.51%
*E. coli*	25	7.55%
*Klebsiella oxytoca*	1	0.30%
*Klebsiella pneumoniae*	7	2.11%
*Morganella morganii*	1	0.30%
*Proteus mirabilis*	10	3.02%
*Pseudomonas aeruginosa*	11	3.32%
*Serratia marcescens*	1	0.30%
*Candida* spp.	5	1.51%
